# Lipid Accumulation Promotes Tumorigenic Behaviour of Colorectal Cancer Cells

**DOI:** 10.1111/jcmm.71393

**Published:** 2026-10-03

**Authors:** Tatjana Itzenhäuser, Fulya Suzan Schildt, Seraphine Jochem, Judith Sommer, Michael Stürzl, Elisabeth Naschberger, Anja Katrin Boßerhoff, Claus Hellerbrand

**Affiliations:** ^1^ Institute of Biochemistry Friedrich‐Alexander‐Universität Erlangen‐Nürnberg Erlangen Germany; ^2^ Division of Molecular and Experimental Surgery, Translational Research Center, Department of Surgery Universitätsklinikum Erlangen, Friedrich‐Alexander‐Universität Erlangen‐Nürnberg Erlangen Germany; ^3^ Comprehensive Cancer Center (CCC) Erlangen‐EMN Erlangen Germany

**Keywords:** colorectal cancer, hyperlipidemia, lipid droplets, lipid metabolism, obesity

## Abstract

Obesity and hyperlipidemia are risk factors for the development and progression of different types of cancer including colorectal cancer (CRC). The underlying mechanisms are only incompletely understood. The aim of this study was to assess the impact of hyperlipidemic conditions on tumorigenic behaviour of CRC cells and the effect of obesity on lipid metabolism in CRC tissues. Oleic acid complexed to albumin was added to the cell culture medium of human colon cancer cell lines HT29, Caco‐2, HCT116, LoVo, SW480 and SW48. This led to a time‐ and dose‐dependent uptake of oleate and triglyceride accumulation and increased expression of perilipin‐2 (PLIN2), a structural component of lipid droplets and carnitine palmitoyltransferase 1 A (CPT1A), the key enzyme of beta‐oxidation. Furthermore, proliferation, migratory activity and the expression of epithelial‐mesenchymal transition (EMT) markers and the proinflammatory cytokine interleukin‐8 (IL‐8) were dose‐dependently increased in CRC cells. In tumorous tissues of obese CRC patients, PLIN2 expression tended to be higher than in tissues of CRC patients with normal weight and immunohistological analysis showed a stronger PLIN2 immunosignal and Ki67‐staining a higher proliferative index in CRC tissues of obese patients. Furthermore, we observed a significant correlation between PLIN2 and CPT1A expression in human CRC tissues and CRC patients with high PLIN2 or CPT1A expression showed a poorer overall and disease‐free survival. In conclusion, our data indicate that fatty acid uptake promotes tumorigenicity of CRC cells. The exogenous uptake of fatty acids could be particularly important in obesity associated hyperlipidemia and herewith may be a critical factor by which obesity promotes CRC progression. Here, these findings support further evaluation of the structural protein PLIN2 as tumour marker as well as therapeutic target in CRC patients.

## 1 Introduction

Colorectal Cancer (CRC) remains a global health problem with yearly increasing incidence [1]. It is the third most common cancer with the second highest mortality rate worldwide [2]. Up to 75% of CRC cases are attributable to modifiable lifestyle factors, such as smoking, excessive consumption of alcohol and red or processed meat, physical inactivity or high fat intake [1, 3]. High calorie intake and a sedentary lifestyle are also the major causes of the increasing prevalence of overweight and obesity, which belong to the most important preventable risk factors for developing CRC [4, 5]. Moreover, it has been demonstrated that obesity is also associated with CRC progression, poor prognosis and higher death rates of CRC patients [6, 7].

Besides systemic alterations of the lipid metabolism, such as obesity, several lines of evidence suggest that lipid metabolism alterations within the tumour cells are critically involved in the pathogenicity of CRC. Lipid metabolism is often reprogrammed in cancer cells which show upregulated de novo synthesis of fatty acids that have been associated with a poor prognosis in neoplastic processes including CRC [8, 9, 10].

Intracellular fatty acids are derived predominantly from neutral lipids stored in lipid droplets. Triglyceride mobilization from lipid droplets occurs through a sequential lipolytic cascade involving adipose triglyceride lipase (ATGL), hormone‐sensitive lipase (HSL) and monoacylglycerol lipase (MAGL). The first rate‐limiting step is catalysed by ATGL, whereby triacylglycerol is converted into diacylglycerol (DAG) and a free fatty acid. Subsequently, HSL catalyses the hydrolysis of DAG to monoacylglycerol (MAG) and a second free fatty acid. In a final step, MAGL hydrolyzes MAG into glycerol and the remaining free fatty acid [11, 12]. The triglyceride accessibility of lipid droplets is regulated by proteins of the perilipin (PLIN) family (PLIN1‐5) [13].

Interestingly, enhanced lipolysis as modulated by elevated expression of MAGL further contributes to high fatty acid levels and CRC progression [14] and thus indicates the high demand of CRC cells on fatty acids. Enhanced ATGL expression has been associated with lipid mobilization and metabolic reprogramming [11, 15]. Further, increased expression was discovered in gastrointestinal cancer and linked to the progression of CRC, highlighting the important role of lipases in CRC metabolism [15, 16]. Lipolysis is further regulated by beta‐adrenergic stimulation activating protein kinase A (PKA) and resulting in the phosphorylation of lipid droplet associated proteins, including perilipins. Thus, the recruitment and activation of lipolytic enzymes at the lipid droplet surface are elevated. PKA‐dependent regulation of perilipins and the associated coactivator CGI‐58 facilitated the mobilization of stored triglycerides and is important for fatty acid availability [13].

The fatty acids released during lipolysis can be used as substrates for energy production via mitochondrial beta‐oxidation. Long‐chain fatty acids are activated to fatty acid acyl‐CoA and transported to the mitochondria via the carnitine shuttle. Carnitine palmitoyltransferase 1A (CPT1A) is a key enzyme of mitochondrial fatty acid utilization and catalyses the conversion of long‐chain fatty acyl‐CoA to acylcarnitine at the outer mitochondrial membrane. This is an important rate‐limiting step in transporting fatty acids into the mitochondria for beta‐oxidation. Therefore, regulation of CPT1A may influence the extent to which cells utilize fatty acids as an oxidative energy source [17].

Lipid metabolism is also transcriptionally regulated. Several signalling pathways and transcription factors have been implicated in the regulation of lipid uptake, storage, lipolysis, fatty acid transport and beta‐oxidation. These include the glucocorticoid receptor, CREB, JAK/STAT signalling, C/EBP family members, PPARalpha/gamma, SREBP, NRF2 and RUNX proteins, for example, CPT1A expression is associated with the signalling pathway JAK/STAT, showing links between oncogenic signalling pathways and fatty acid utilization [18]. Furthermore, upregulated glucose uptake is fueling lipogenesis [8].

In addition to lipolysis and de novo synthesis, exogenous uptake is also an important source for fatty acids. In particular, during metabolic stress, cancer cells adapt to their environment [19]. Notably, obesity is frequently associated with high circulating levels of fatty acids [20] leading to the hypothesis that exogenous fatty acid uptake contributes to the tumour promoting effects of obesity in CRC patients.

In the present study, we used an in vitro model mimicking the condition of high circulating levels of fatty acids as found particularly in obese patients. We observed an uptake of the fatty acids and consecutive triglyceride accumulation and lipid droplet formation in human CRC cells in vitro and analysed the effect of the lipid accumulation on the tumorigenicity of the tumour cells. Moreover, we analysed the correlation between lipid droplets in tumorous tissue, the body mass index (BMI) and the prognosis of CRC patients.

## 2 Material and Methods

### 2.1 Cell Culture and Induction of Cellular Lipid Accumulation

The human CRC cell lines HT29 (ATCC HTB‐38), Caco‐2 (ATCC HTB‐37), SW48 (ATCC CCL‐231), SW480 (ATCC CCL‐228), HCT116 (ATCC CCL‐247) and LoVo (ATCC CCL‐229) were cultured in DMEM supplemented with penicillin (400 U/mL), streptomycin (50 μg/mL), L‐glutamine (5840 mg/L), glucose (450 mg/dL) and 10% fetal calf serum (FCS) and incubated at 37°C in an 8% CO_2_ atmosphere as described in [21].

To induce cellular lipid accumulation, cells were seeded in standard 6‐well‐plates at a density of 4.5 × 10^5^ cells/well in DMEM containing 100 mg/dL of glucose and were allowed to adhere for 24 h before treatment. Cells were incubated with oleic acid (Sigma, Steinheim, Germany) complexed to bovine serum albumin (BSA; Roth, Wernberg‐Köblitz, Germany) at the indicated time points and concentrations as described [22].

### 2.2 Human Tissues

Tissues from patients with CRC were used for RNA and immunohistological analyses. This was approved by the ethics committee (institutional review board) of the Friedrich‐Alexander Universität (FAU) Erlangen‐Nürnberg (approval number: 3914, Polyprobe study). All participants were informed personally and provided written informed consent for this study. Patient data were pseudonymized and all analyses were performed in accordance with the Helsinki declaration.

### 2.3 Analysis of Lipid Content

Oil red O staining of intracellular lipids was performed as described [22] with slight modifications. In brief, oleate treated cells and control cells were fixed with 4% paraformaldehyde (PFA) for 5 min, washed with 60% isopropanol and incubated with oil red O (Sigma, Steinheim, Germany) for 45 min at room temperature. Cells were washed with H_2_O and subsequently examined by microscopy. Triglycerides were extracted and analysed using the triglyceride‐hit (PAP) kit (BIOMED, Oberschleißheim, Germany) as described previously [23]. Concentrations of free fatty acids were analysed using the Free Fatty Acid Assay Kit (Abcam, ab65341, Cambridge, UK) according to the manufacturer's instructions.

### 2.4 Analysis of Cell Proliferation

Cell proliferation was measured using the colorimetric XTT assay (Roche Diagnostics, Mannheim, Germany) and following the manufacturer's instructions as described before [22].

### 2.5 Analysis of mRNA Expression

RNA was isolated from cultured cells and subsequent reverse transcription was performed as described [22]. Quantitative real‐time‐PCR was performed applying LightCycler technology (Roche, Mannheim, Germany). The following sets of primers were used: ACLY (forward: *5′‐CGG ACT TCG GCA GAG GTA GA‐3′*, reverse: *5′‐GGG GAG GGA ACT CGA TGT CA‐3′*); CPT1A (forward: *5′‐CCC GGG GGA ATG TCA AGA GG‐3′*, reverse: *5′‐CGC ACG AAG TCG CAT GAC TC‐3′*); DGAT2 (forward: *5′‐CTC ATA GCC GCC TAC TCC G‐3′*, reverse: *5′‐CAA GGA AGG ACA GGA CCC AC‐3′*); E‐CAD (forward: *5′‐TAA CCG ATC AGA ATG AC‐3′*, reverse: *5′‐TTT GTC AGG GAG CTC AGG AT‐3′*); FASN (forward: *5′‐ACA CAC GAT GGA CCC TCA GC‐3′*, reverse: *5′‐CAC GAG TGT CTC GGG GTC TC‐3′*); HSL (forward: *5′‐GTT AAG TGG GCG CAA GTC CC‐3′*, reverse: 5′‐GGT GTG GAG GGG CTA AGT GT‐3′); IL‐8 (forward: *5′‐TCT GCA GCT CTG TGT GAA GGT GCA GTT‐3′*, reverse: *5′‐AAC CCT CTG CAC CCA GTT TTC CT‐3′*); MAGL (forward: *5′‐TGT GGA ATG CAA ACG CCA GC‐3′*, reverse: *5′‐AGC ATC CGA GCC AGC TCT TC‐3′*); N‐CAD (forward: *5′‐TGG ATG AAG ATG GCA TGG‐3′*, reverse: *5′‐AGG TGG CCA CTG TGC TTA C‐3′*); PLIN1 (forward: *5′‐CCA ACA CCC TCT CTC GAT ACA CC‐3′*, reverse: *5′‐GGG CTG CTA CCT CAC TGA ACT T‐3′*); PLIN2 (forward: *5′‐GCT GCA GTC CGT CGA TTT CTT T‐3′*, reverse: *5′‐CCA CAC TCG GTT GTG GAT CA‐3′*); PLIN3 (forward: 5′‐GGA GAG CTA CCC GCA CAT CA‐3′, reverse: 5′‐ACA CCT TAG ACG ACA CAA GCT CC‐3′); PLIN4 (forward: 5′‐GGC ATT TGA ACA CGC GGT GAG‐3′, reverse: 5′‐GGC ATT TGA ACA CGC GGT GAG‐3′); PLIN5 (forward: 5′‐GGT GGA TCA CTT CCT GCC CAT‐3′, reverse: 5′‐CTC TGA TCC TCC ACC GAA CCC‐3′); SCD1 (forward: *5′‐TGC CCC TAC GGC TCT TTC TG‐3′*, reverse: *5′‐TTG ACA GCT GGG TGT TTG CG‐3′*); SNAIL (forward: *5′‐ACT ATG CCG CGC TCT TTC CT‐3′*, reverse: *5′‐GCT GCT GGA AGG TAA ACT CTG G‐3′*); TWIST (forward: *5′‐CGG ACA AGC TGA GCA AGA TT‐3′*, reverse: *5′‐CCT TCT CTG GAA ACA ATG AC‐3′*). Amplification of cDNA derived from human 18S rRNA (forward: *5′‐TCT GTG ATG CCC TTA GAT GTC C‐3′*, reverse: *5′‐CCA TCC AAT CGG TAG TAG CG−3′*) was used for normalization of data.

RNA isolation from human CRC tissue was conducted as described [24]. In brief, RNA was isolated using a fully automated extraction method from FFPE tissue (Tissue Preparation System with VERSANT Tissue Preparation Reagents, Siemens Healthcare Diagnostics, Tarrytown, NY). Paraffin sections (10 μm, tumour content > 30%) were directly subjected to automated total nucleic acid extraction. Samples were heat‐lysed in 150 μL FFPE buffer at 80°C for 30 min with shaking. After cooling, enzymatic lysis was carried out at 65°C for 30 min with proteinase K. Any residual tissue debris was removed by nonspecific binding to silica‐coated iron‐oxide beads and subsequent magnetic separation. Deparaffinized and clarified lysates were transferred to new tubes and nucleic acids were bound to fresh silica‐coated beads under chaotropic conditions. Beads were washed three times and total nucleic acids were eluted with 100 μL of elution buffer at 70°C followed by an automated DNase I digestion.

RT‐qPCR analysis from human CRC tissue was conducted as described [24]. In brief, RT‐qPCR primers and 5´‐FAM‐3´‐BHQ‐1‐labelled probes were designed using PrimerBlast (NCBI). The primers and probes were purchased by Eurogentec (Serain, Belgium). The RNA primer/probe sequences were PLIN2_FFPE (forward: *5′‐GCT GCA GTC CGT CGA TTT CT‐3′*, probe: *5′‐CCA GGA AGA AAA ATG GCA TCC GTT GC‐3′*, reverse: *5′‐CCA CAC TCG GTT GTG GAT CA‐3′*) and RPL37A (housekeeper; forward: *5′‐TGT GG TTC CTG CAT GAA GAC A‐3′*, probe: *5′‐TGG CTG GCG GTG CCT GGA‐3′*, reverse: *5′‐GTG ACA GCG GAA GTG GTA TTG TAC‐3′*). The DNA primer/probe sequences were PAEP (forward: *5′‐CAC AGA ATG GAC GCC ATG AC‐3′*), probe*: 5′‐AAG CCC TCA GCC CTG CTC TCC ATC‐3′*, reverse: *5′‐AAA CCA GAG AGG CCA CCC TAA‐3′*.

The SuperScript III Platinum One‐Step Quantitative RT‐PCR‐System with ROX (Life Technologies) was used as recommended by the manufacturer except for a prolonged reverse transcription time of 30 min at 50°C. A reaction (10 μL total volume) consisted of 0.2 μL RT/Taq‐mix, 50 nM ROX Reference Dye (Life Technologies), 10 ng total RNA, 500 nM forward/reverse primer each and 250 nM probe. The reactions were assayed in triplicates in 96‐well‐qPCR‐plates (Agilent Technologies, Santa Clara, CA, USA) using a Mx3005P qPCR system (Agilent) together with the Versant kPCR software (Siemens Healthcare Diagnostics). For quantification of RNA samples the absence of residual DNA was analysed by DNA‐specific primers for the progestogen‐associated endometrial protein (PAEP) gene and was in all cases negative. On each plate a commercial qPCR human reference total RNA (qRef, Agilent) was used as a positive control and a non‐template control (NTC) as negative control. The fluorescence threshold (ROX dRn) was set to 0.02 for all samples. All samples were normalized using RPL37A (mean of triplicates) as a reference gene. Subsequently, the ΔΔCtmethod was used for calculation of the respective fold changes for each target gene.

### 2.6 Analysis of Migratory Activity of Colon Cancer Cells

Migratory activity of colon cancer cells was assessed using Boyden chambers and a Boyden chamber‐based assay (Abcam, ab235697, Cambridge, UK) according to the manufacturer's instructions without the use of the basement membrane matrix.

### 2.7 Immunohistochemistry and Immunofluorescence Staining

For immunohistochemical analysis standard sections of formalin‐fixed and paraffin‐embedded tissue blocks were used. Staining was performed as described [25] using the anti‐PLIN‐2 antibody (HPA016607, Sigma, Steinheim, Germany 1:100) and anti‐Ki67 antibody (ab16667, Abcam, 1:500, Cambridge, UK). For immunofluorescence staining, cells were fixed with 4% PFA for 10 min and permeabilized in 0.5% TritonX100. After that, cells were blocked in 1% BSA. Anti‐PLIN‐2 antibody was incubated overnight at 4°C (1:200). Alexa Fluor 546‐conjugated donkey anti‐rabbit IgG (A11010, Thermo Fisher Scientific, Waltham, MA, USA) was used as secondary antibody for 1 h at 37°C.

### 2.8 Western Blot

Protein isolation and western blot analysis were conducted as previously described [23] using anti‐PLIN2 antibody (15294‐1‐AP, Proteintech, Rosemont, IL, USA 1:2000), anti‐CPT1A (15184‐1‐AP, Proteintech, Rosemont, IL, USA, 1:1000), anti‐ATGL (55190‐1‐AP, Proteintech, Rosemont, IL, USA 1:1000) and anti‐ACLY (EP704Y, Abcam, Cambridge, UK, 1:1000). As a secondary antibody, mouse‐anti rabbit (sc‐2357, Santa Cruz Biotechnology, Heidelberg, Germany 1:10000) was used.

### 2.9 Seahorse Analysis

For the Seahorse Mito Stress Test analysis, 15,000 cells/well were seeded in XFp microplates (Agilent Technologies Inc., Santa Clara, CA, USA) and oleate treatment was carried out as described above. Mito Stress test and analysis was performed as described [26] with the first step being an additional injection of etomoxir (4 μM) and an appropriate control.

### 2.10 Statistical Analysis

Values are presented as mean ± SEM. Comparison between groups was made using the student's unpaired *t*‐test or one‐way ANOVA for multiple comparison when appropriate. *p* < 0.05 was considered statistically significant. All calculations were performed using the GraphPad Prism version 6.01 for Windows (GraphPad Software, San Diego, CA, USA).

## 3 Results

### 3.1 Incubation With Fatty Acids Induces Cellular Lipid Accumulation in Human Colon Cancer Cell Lines

Obesity and the metabolic syndrome, respectively, are characterized by hyperlipidemia. Free fatty acids reaching the circulation from adipose tissue are transported in the blood bound to albumin. We applied an in vitro model simulating these conditions in the human CRC lines HT29, Caco‐2, SW480, SW48, LoVo and HCT116. Our study comprised three MSS (HT29, Caco‐2, SW480) and three MSI (SW48, LoVo, HCT116) cell lines with distinct molecular backgrounds and driver mutations (Supplementary Table 1; [27]). Here, oleic acid complexed to albumin was added to the cell culture medium. Incubation with serial dilutions of oleate for 48 h induced a dose‐dependent accumulation of intracellular triglycerides in HT29 and Caco‐2 cells (Figure 1A). Enhanced formation of cytosolic lipid droplets was evidenced by oil red O staining (Figure 1B). In parallel, fatty acid concentration in the supernatant declined with time indicating a time‐dependent uptake of fatty acids by the HT29 cells (Figure 1C). Fitting to this, mRNA and protein expression levels of perilipin‐2 (PLIN2), a structural component of lipid droplets, rose dose‐dependently in cancer cells treated with oleate compared to control cells (Figure 1D). Similar findings were also observed in the other cell lines (HCT116, LoVo, SW480 and SW48) (Supplementary Figure 1A). Other members of the perilipin family were also evaluated. While *PLIN4* and *PLIN3* show the same trend, the other members (*PLIN1* and *PLIN5*) showed no significant induction of mRNA expression (Supplementary Figure 1B). We focused on PLIN2, as it showed the most pronounced and consistent response and because of its described relevance in fatty acid uptake. Immunofluorescence (IF) staining confirmed induced protein levels of PLIN2 visualizing the enhanced formation of lipid droplets in the cytoplasm of cancer cells after incubation with oleic acid (Figure 1E). In summary, these findings indicate the uptake of fatty acids and their storage in lipid droplets in the form of triglycerides in CRC cells.

**FIGURE 1:**
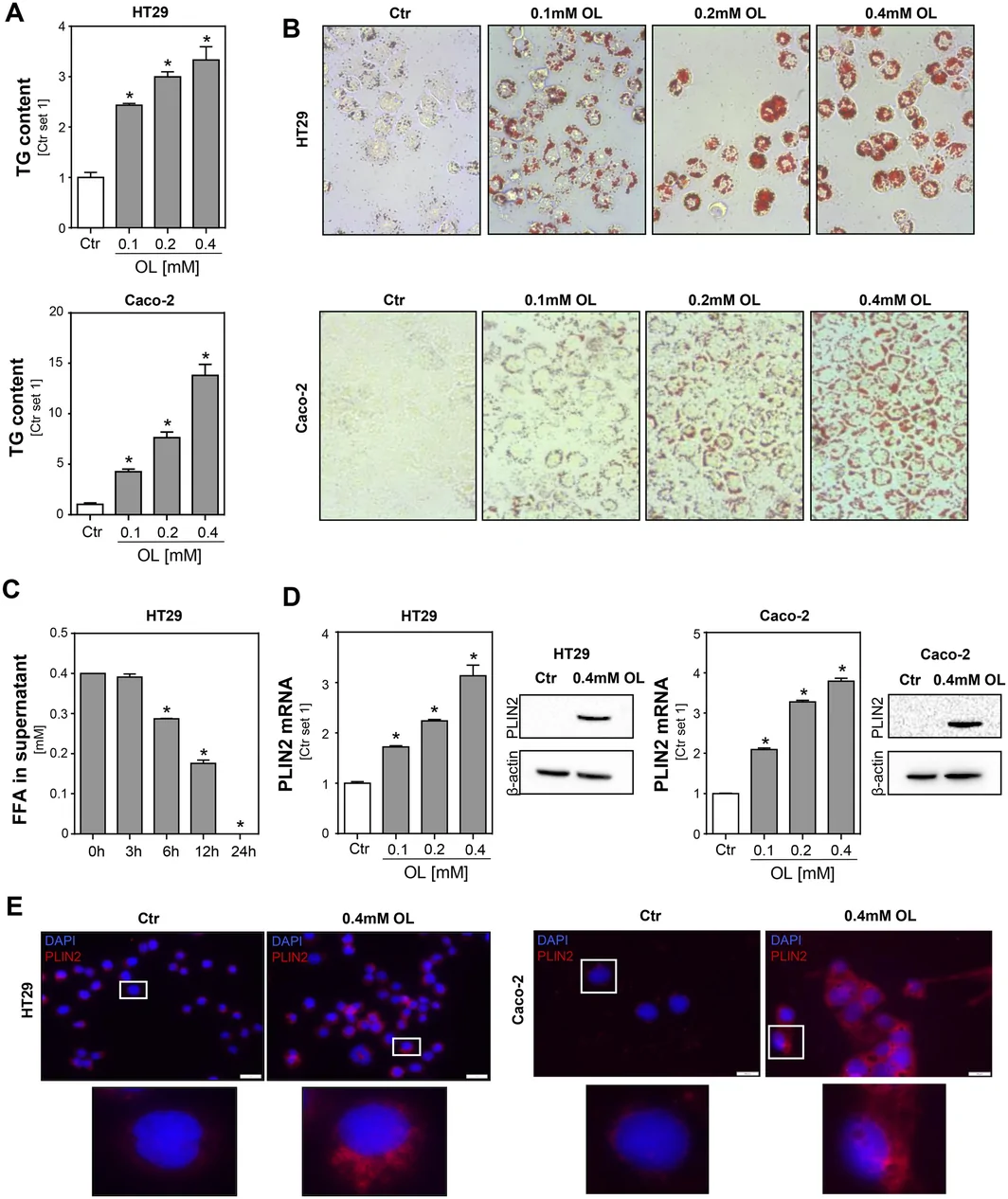
Effect of oleate treatment on cellular lipid accumulation in CRC cells. HT29 and Caco‐2 cells were incubated with indicated oleate (OL) concentrations for 3–48 h. (A) Intracellular triglyceride levels were normalized to cellular protein content. (B) Representative light microscopic images of oil red O staining (400× magnification). (C) Free fatty acids in the supernatant of cells incubated with 0.4 mM OL at indicated time points. (D) PLIN2 mRNA expression analysed by quantitative RT‐PCR and protein expression analysed by western blot. (E) Representative images of immunofluorescence staining for PLIN2 (red) (Magn. 400×). Cell nuclei were stained with DAPI (blue) (*p < 0.05 compared to control (Ctr)).

### 3.2 Effect of Lipid Uptake on Expression of Key Enzymes of Lipid Metabolisms in Cancer Cells

The uptake of oleate leads to a slight but significant reduction of the mRNA expression of *ATP citrate synthase* (*ACLY*), which generates cytosolic acetyl‐CoA and oxaloacetate from citrate and CoA from aerobic glycolysis and herewith, provides the building blocks for de novo lipid synthesis. Furthermore, ACLY protein levels also showed a trend toward reduced expression in HT29 and Caco‐2 cells (Figure 2A; Supplementary Figure 1C,2A). In line with this, we observed a dose‐dependent reduction of the mRNA expression of *fatty acid synthase* (*FASN*), the multi‐enzyme protein that catalyses fatty acid synthesis, in oleate‐stimulated CRC cells (Figure 2B; Supplementary Figure 2B). Furthermore, the mRNA expression of *stearoyl‐CoA desaturase 1 (SCD1)*, the key enzyme for desaturation of saturated fatty acids, was reduced fitting to the supply and uptake, respectively, of the monounsaturated oleic acid (Figure 2C; Supplementary Figure 2C). In contrast and fitting to the elevated triglyceride levels, the mRNA expression of *diacylglycerol‐O‐acyltransferase 2 (DGAT2)*, a key enzyme of triglyceride synthesis, was increased in HT29 and Caco‐2 cells after oleate uptake (Figure 2D). However, *DGAT2* mRNA expression was not altered in HCT116 and SW480 cells and showed only a slight, non‐significant, increase in LoVo and SW48 cells (Supplementary Figure 2D). Furthermore, the mRNA expression of *carnitine palmitoyltransferase IA (CPT1A)*, the key enzyme of beta‐oxidation, increased dose‐dependently in cells cultured with oleate in the supernatant. In line with this, protein levels of CPT1A also increased upon oleate treatment (Figure 2E; Supplementary Figure 3A). To directly investigate the combustion of fatty acids, we performed a mitochondrial stress test (Seahorse assay) in HT29 and Caco‐2 cells with etomoxir added prior to the sequential injection of oligomycin, FCCP and rotenone/antimycin A. We analysed basal respiration prior to the inhibitor injections and determined maximal respiration after FCCP injection. Oleate addition showed a slight increase in basal respiration compared to control cells, however these effects did not reach statistical significance. In HT29 cells, oleate also showed non‐significant increase in maximal respiration. Etomoxir injection resulted in a reduced maximal respiration in both control and oleate treated HT29 cells, suggesting that inhibiting oleate fatty acid oxidation affects mitochondrial respiration. In contrast, Caco‐2 cells showed comparable levels of maximal respiration in control and oleate treated cells, irrespective of etomoxir treatment (Supplementary Figure 3B). ATGL, the key enzyme of intracellular triglyceride hydrolysis, was investigated following oleate supplementation. ATGL protein levels increased in HT29, Caco‐2, HCT116, SW480 and SW48 cells, but not in LoVo cells (Figure 2F; Supplementary Figure 3C). The mRNA expression levels of the two additional lipolytic enzymes HSL (encoded by *LIPE*) and MAGL (encoded by *MGLL*) also increased after oleate uptake in HT29 cells (Supplementary Figure 3D).

**FIGURE 2:**
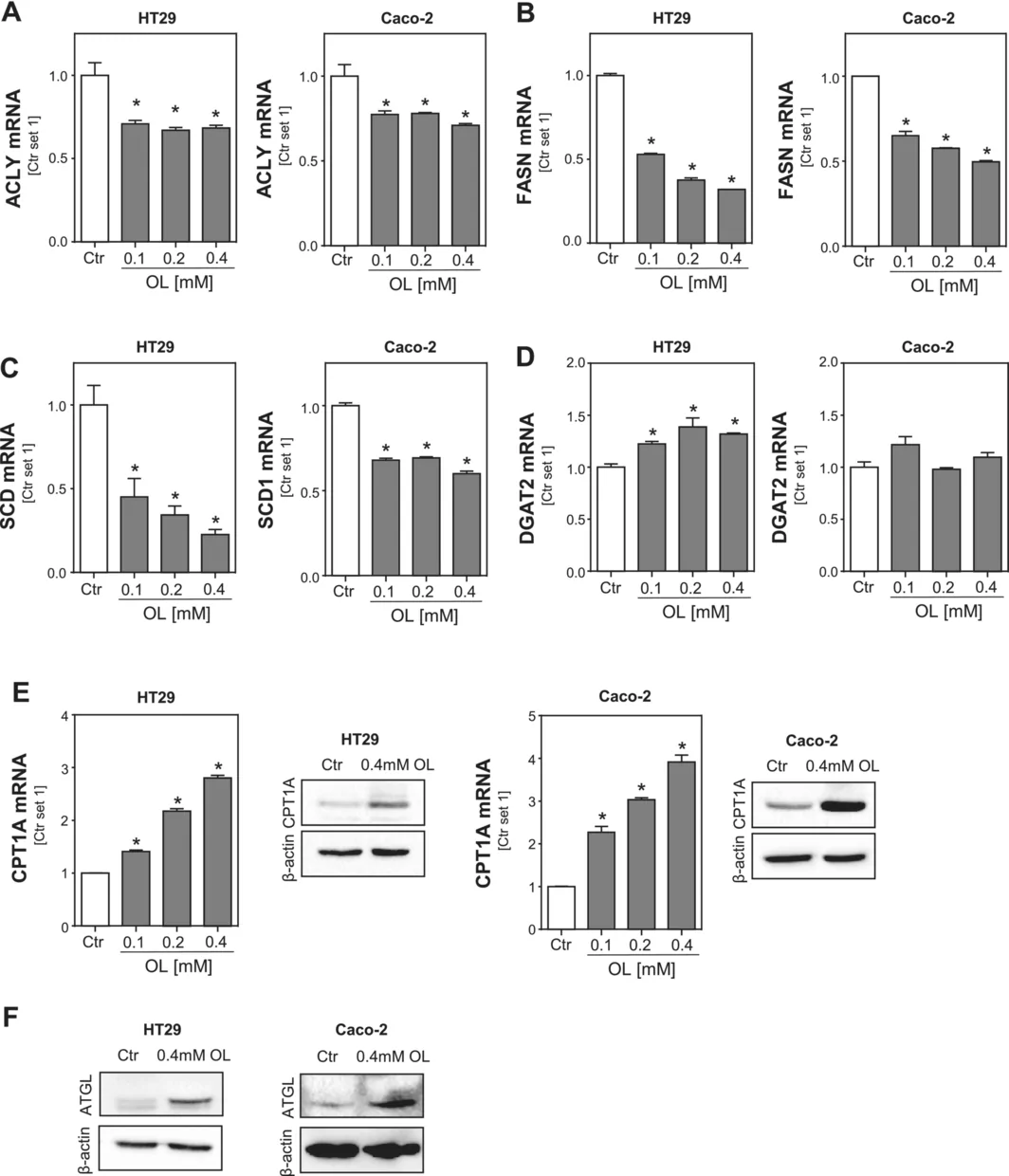
Effect of lipid uptake on expression of key enzymes of lipid metabolism in CRC cells. HT29 and Caco‐2 cells were treated with indicated oleate (OL) concentrations for 12–24 h. (A) ACLY mRNA expression. (B) FASN mRNA expression. (C) SCD1 mRNA expression. (D) DGAT2 mRNA expression and (E) CPT1A mRNA expression analysed by quantitative RT‐PCR and CPT1A protein expression analysed by western blot. (F) ATGL protein expression analysed by western blot (*p < 0.05 compared to control (Ctr)).

These data indicate that the external supply of fatty acids in part takes over the increased supply of fatty acids for cancer cells instead of de novo lipogenesis. Still, in sum, the external supply leads to both increased storage of fatty acids in the form of triglycerides as well as fatty acid combustion and energy generation by beta‐oxidation.

### 3.3 Effect of Cellular Lipid Accumulation on Tumorigenicity of Cancer Cells

Next, we aimed to analyse whether the oleate uptake has functional impact on the tumorigenicity of CRC cells. We determined that the proliferation of CRC cells was dose‐dependently increased in oleate‐stimulated cells (Figure 3A; Supplementary Figure 4A). Moreover, oleate‐stimulated cells showed enhanced migratory activity in Boyden chamber assays (Figure 3B; Supplementary Figure 4B). Fitting to this, oleate stimulation also led to a downregulation of *E‐cadherin (E‐Cad)* mRNA expression and in parallel, to increased *N‐cadherin (N‐Cad)* mRNA expression (Figure 3C). This “cadherin switch” is linked to greater migratory and invasive capabilities. The loss of E‐cadherin destabilizes the adhesive junctions between epithelial cells, facilitating cancer cells to detach from the primary tumour site [28]. Furthermore, the expression of the epithelial‐mesenchymal transition (EMT) markers *SNAIL* and *TWIST* was increased in oleate stimulated HT29 cells (Figure 3D,E). In contrast, EMT markers were only slightly altered in oleate treated Caco‐2 and LoVo cells and not significantly changed in HCT116, SW480 and SW48 cells (Figure 3C–E; Supplementary Figure 5A–D). However, the expression of *CXCL8* (Interleukin‐8 (IL‐8)), a proinflammatory marker associated with poor outcome of patients with colon carcinoma [29], increased dose‐dependently in HT29, Caco‐2, HCT116 and LoVo cells incubated with oleate, while SW480 and SW48 showed no significant changes (Figure 3F; Supplementary Figure 6A). Together, these data indicate that the uptake of oleate increases the in vitro tumorigenicity of CRC cells.

**FIGURE 3:**
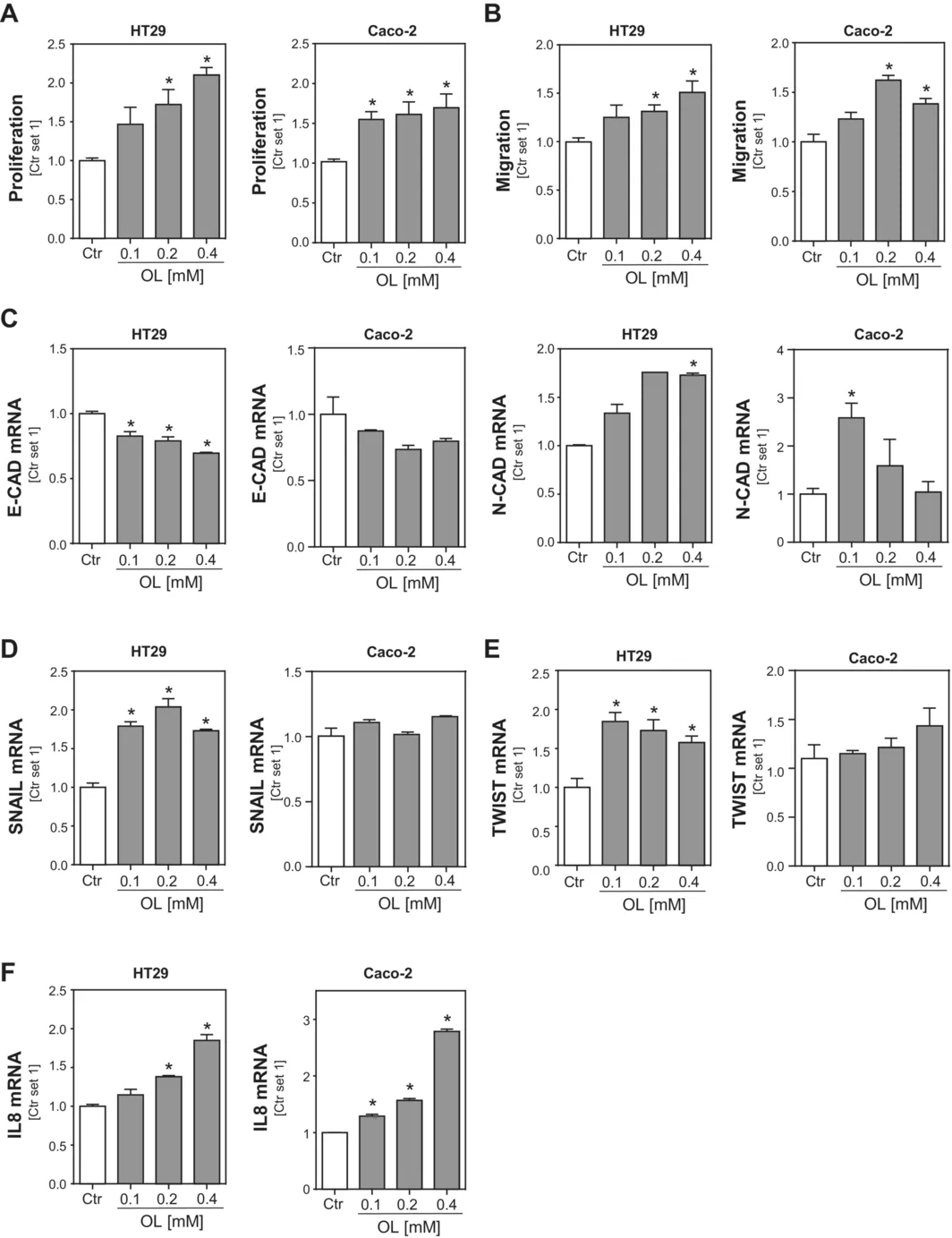
Effect of cellular lipid accumulation on tumorigenicity of cancer cells. HT29 and Caco‐2 cells were treated with indicated oleate (OL) concentrations for 12–24 h. (A) Proliferation and (B) migratory activity of CRC cells. mRNA expression of EMT‐markers (C) E‐CAD and N‐CAD and (D) SNAIL (E) and TWIST. (F) mRNA expression of IL‐8 (*p < 0.05 compared to control (Ctr)).

### 3.4 PLIN2 and CPT1A Expression in Tumorous Tissue of Patients With Colorectal Carcinoma

We have found in vitro that elevated fatty acid levels in the supernatant lead to fatty acid uptake and increased PLIN2 expression in CRC cells. Most obese patients have elevated fatty acid levels in the serum. Therefore, we analysed PLIN2 expression in tumorous tissues of patients with CRC and either normal weight (BMI: 20–25 kg/m^2^; *n* = 14) or obesity Grade I or II (BMI: 30–40 kg/m^2^; *n* = 11). Obese patients showed a tendency to higher PLIN2 expression, although differences did not reach the levels of significance (Figure 4A). From 10 of these patients also tissue for immunohistological (IH) analysis was available. IH‐staining for PLIN2 showed a strong immunosignal in tissues of obese patients, while the staining intensity was weaker in tissues of patients with normal weight (Figure 4B). Moreover, Ki67‐IH showed that the number of positive cells was higher in CRC‐tissues of obese patients compared to patients with normal weight (Figure 4C). Interestingly, in silico analysis showed that high PLIN2 expression correlated with poor overall and disease‐free survival in different cohorts of patients with colorectal carcinoma (Figure 4D; Supplementary Figure 6B). Furthermore, we observed a significant correlation (*r* = 0.37; *p* = 4e‐10) between PLIN2 expression and CPT1A expression in tissues of patients with CRC (Figure 4E) and patients with high CPT1A expression showed a poorer overall and disease‐free survival (Figure 4F; Supplementary Figure 6C). Together, these findings indicate that PLIN2 expression is higher in obese patients and that—similar as in vitro—the higher PLIN2 expression correlates with higher CPT1A expression and a poorer clinical outcome.

**FIGURE 4:**
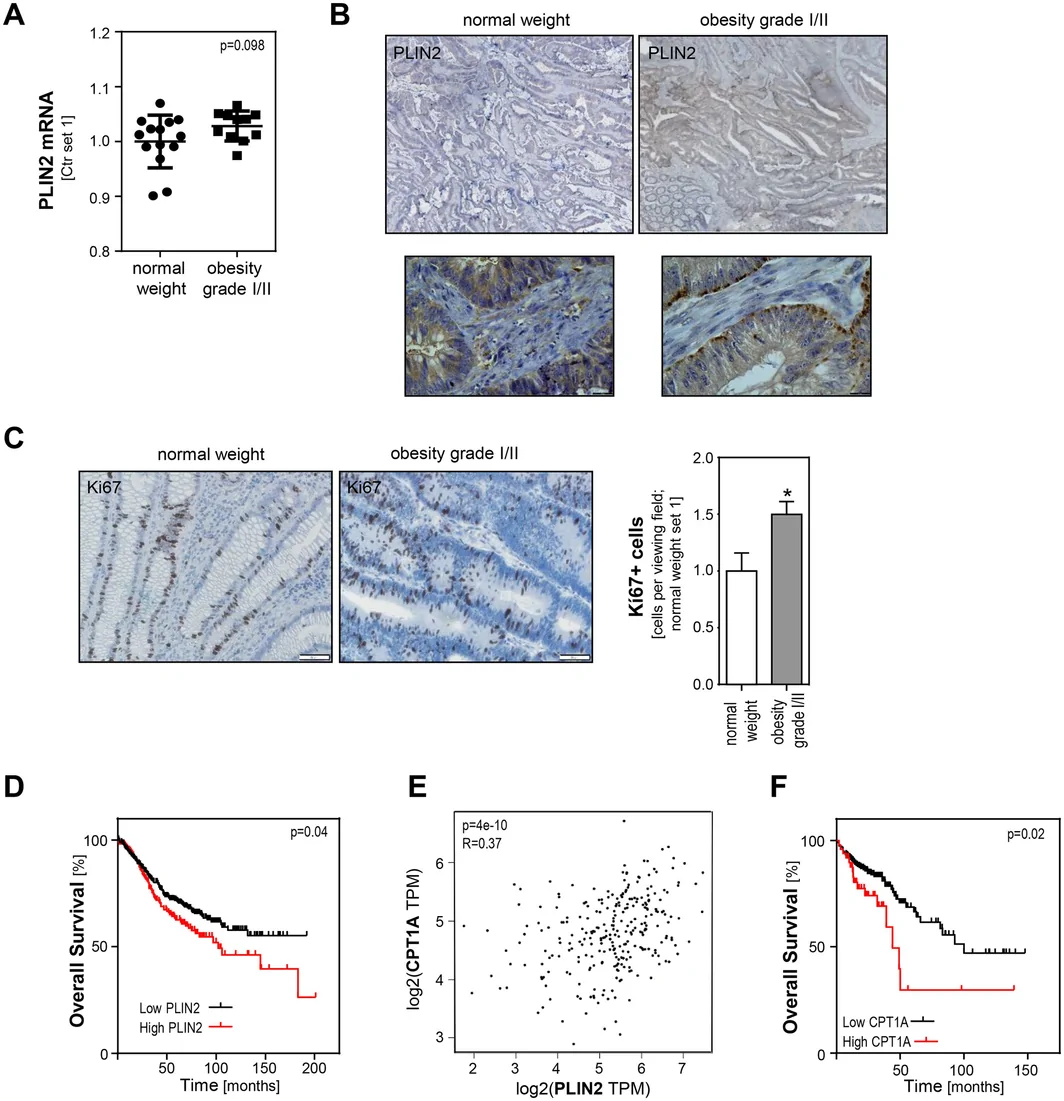
PLIN2 and CPT1A expression in tumorous tissue of patients with colorectal carcinoma. Analysis of CRC tissue samples from patients with either normal weight (BMI: 20–25 kg/m^2^; *n* = 14) or obesity Grade I or II (BMI: 30–40 kg/m^2^; *n* = 11). (A) mRNA expression of PLIN2. (B) Representative images of immunohistological (IH) staining of PLIN2 (Magn. 20×) and higher resolution of same region (Magn. 630×). (C) Representative images of IH staining of Ki67 (left panel (Magn. 100×)); number of Ki67‐positive cells per viewing field (right panel). (D) Correlation between high/low PLIN2 expression and overall survival of CRC patients applying a Marisa Colon GSE40967‐GPL570 dataset of 579 patients and SurvExpress database. (E) Correlation of CPT1A and PLIN2 mRNA expression levels (log2(transcript per million)) in 350 human CRC tissues. The cancer genome atlas (TCGA)‐derived data were used, applying the gene expression profiling interactive analysis (GEPIA) database. (F) Correlation between high/low CPT1A expression and overall survival of CRC patients applying a TCGA dataset of 350 patients and SurvExpress database (*p < 0.05 compared to control (Ctr)).

## 4 Discussion

Since hyperlipidemia is a major feature of obesity and the metabolic syndrome, we used an in vitro model to study the molecular mechanisms linking high fatty acids found in the circulation of patients with metabolic alterations and tumorigenicity of CRC cells. Plasma free fatty acid concentration is elevated in obesity, reaching concentrations in the range used in our in vitro study [30, 31, 32]. The free form of fatty acids is primarily derived from lipolysis of triglycerides and free fatty acids circulate predominantly bound to albumin in the bloodstream [33]. Oleic acid represents approximately 80% of the circulating monounsaturated fatty acids and therefore served as the primary exposure variable in the present study [34]. Importantly, our in vitro model does not recapitulate complete obesity‐related hyperlipidemia, since this is a more complex process involving multiple lipids, adipokines, hormonal signalling, inflammation and other metabolic factors. Instead, our experimental design focused on the specific contribution of increased extracellular fatty acids to CRC lipid metabolism and progression.

Applying this model, we found that incubation with fatty acids caused significant triglyceride accumulation and the formation of lipid droplets in CRC cells.

Increased number of lipid droplets has been observed in CRC [35] and an increase in the number of lipid droplets has been shown to be associated with cell cycle progression in CRC cell lines [36]. In line with this, we observed that the uptake of exogenous fatty acids induced the proliferation of CRC cells. Moreover, the migratory activity of CRC cells increased in a dose‐dependent manner upon incubation with fatty acids. These findings are supported by previous literature describing the effects of exogenous fatty acids on lipid accumulation, proliferation and migratory phenotypes in several cancer models [37, 38, 39]. Our data extend previous findings by investigating six different CRC cell lines with a distinct molecular background and observing comparable effects of lipid accumulation across these cell lines, suggesting that the cellular response is not restricted to distinct molecular subtypes, driver mutations and is independent of MSS or MSI background.

The proinflammatory cytokine IL‐8 is upregulated in CRC and has been shown to be associated with proliferation, angiogenesis and metastasis [40]. Furthermore, high IL‐8 levels correlated with shorter overall and progression‐free survival of CRC patients [29]. In line with this, increased lipid droplet numbers have been implicated in the production of proinflammatory mediators in CRC cell lines [36]. In our study, we observed changes of mRNA expression of inflammatory and also EMT‐associated genes suggesting that lipid accumulation may be linked to broader transcriptional changes in CRC. The observed increased migration supports this finding, but further evaluation in broader studies is required to define invasion, matrix degradation, metastasis or in vivo spread.

The uptake of exogenous fatty acids also led to altered expression of several key enzymes of lipid metabolism in CRC cell lines. Thus, the expression of CPT1A was increased on protein and mRNA levels. Furthermore, we observed a correlation between PLIN2 and CPT1A expression in human CRC tissues. This association is consistent with regulation of fatty acid metabolism in CRC tissue. Here, further experiments are needed to establish a causal relationship between PLIN2 and CPT1A and to more precisely define their functional roles in regulating the progression of CRC. CPT1A converts fatty acids to acylcarnitines, which are shuttled into mitochondria, where they undergo beta‐oxidation and produce energy. Mitochondrial stress tests in HT29 and Caco‐2 cells showed a slight increase in basal respiration, although these effects did not reach statistical significance. Interestingly, using etomoxir to inhibit CPT1 activity resulted in reduced maximal respiration in both control and oleate‐treated HT29 cells, suggesting that CPT1‐dependent processes contribute to mitochondrial respiratory capacity in these cells. In contrast, Caco‐2 cells exhibited no comparable maximal respiration irrespective of oleate or etomoxir treatment. Thus, the seahorse experiments did not provide consistent evidence of a significant increase in mitochondrial fatty acid oxidation across CRC models. One possible explanation is that increased extracellular fatty acids promote lipid storage rather than immediate oxidative utilization. Indeed, we observed pronounced triglyceride accumulation and lipid droplet formation. Elevated CPT1A expression may therefore reflect an enhanced capacity or an adaptive response to increased fatty acid availability, while most fatty acids still might be stored in lipid droplets. Whether these stored lipids are subsequently mobilized for mitochondrial oxidation may depend on the cellular context and the balance between lipid uptake, triglyceride accumulation, lipolysis and fatty acid oxidation.

Triglyceride hydrolysis is initially catalysed by ATGL, followed by HSL and MGL [12]. ATGL protein levels increased upon oleate treatment in most CRC cell lines. In HT29, the expression of the additional lipolytic enzymes HSL and MGL was also elevated at mRNA levels. These findings indicate that increased lipid storage is accompanied by an adaptation of the intracellular machinery involved in lipid mobilization. However, these changes in expression do not necessarily translate into increased lipolytic activity; further studies are needed to confirm increased lipolytic activity and to investigate the functional contribution of these enzymes to the observed phenotypes.

In general, perilipins are important regulators of lipid droplet biology and are involved in lipid storage and mobilization. The PLIN family comprises several lipid droplet‐associated proteins. While PLIN1 is predominantly expressed in adipose tissue, PLIN2 is more broadly expressed as well in non‐adipocytic cells and is strongly associated with lipid droplet formation and neutral lipid storage [13]. PLIN2 showed a strong increase upon oleate treatment and the most consistent results in our study; therefore we focused mainly on PLIN2. PLIN3 is widely expressed and described in lipid droplet biogenesis and trafficking, whereas PLIN4 is mainly associated with adipose lipid storage. PLIN5 is enriched in oxidative tissues such as muscle or heart tissues and linked to interactions between lipid droplets and mitochondria [13]. We observed no induction of PLIN1 and PLIN5 mRNA expression, but not unexpected given their distinct tissue distribution and functional specialization. Moreover, in line with the results for PLIN2, we observed enhanced expression of PLIN3 and PLIN4 mRNA.

These results are also consistent with PLIN proteins regulating the accessibility of stored lipids to lipases. In particular, PLIN2 has been associated with protection of lipid droplets from lipolysis [41]. Thus, increased PLIN2 expression may contribute to the stabilization of newly formed lipid droplets.

The activity of lipolytic enzymes is not only regulated by their abundance but also by lipid droplet‐associated proteins and post‐translational mechanisms. In adipocytes, beta‐adrenergic stimulation increases intracellular cAMP that activates PKA, which subsequently phosphorylates PLIN1 and HSL [18]. Phosphorylation of PLIN1 promotes the release of its interaction with CGI‐58, allowing it to activate ATGL and thereby facilitating triglyceride hydrolysis [42]. This pathway illustrates how extracellular hormonal signals can rapidly switch from lipid storage to mobilization, showing that it is a very complex system involving many factors and regulators. Whether similar regulatory pathways are involved in our observed phenotype remains unclear. Nevertheless, the strong accumulation of lipid droplets together with increased expression of perilipin proteins may show a similar state of increased lipid storage accompanied by enhanced capacity for lipid mobilization, allowing cells the ability to adapt to metabolic demands.

Fatty acid beta‐oxidation plays a critical role in tumour growth [43, 44] and CPT1 inhibitors have been tested for anti‐cancer effects in different tumour entities including CRC [45]. It might be interesting to analyse whether the efficacy of anti‐CPT1 strategies is affected by exogenous fatty acid uptake or varies in obese and normal weight patients, respectively. Recent work in pancreatic ductal adenocarcinoma (PDAC) demonstrated a functional connection between PLIN2 and CPT1A in regulating lipid droplet and mitochondrial interactions and lipid mobilization under nutrient stress [46]. Further, studies in gastric cancer have linked CPT1A associated metabolic reprogramming to JAK/STAT3 signalling [47]. These findings indicate that similar metabolic changes occur across gastrointestinal tumour entities and specific regulatory mechanisms are likely to be tissue‐ and context‐dependent. The regulation of fatty acid metabolism is complex and involves several transcriptional as well as post‐translational mechanisms. Signalling pathways such as JAK/STAT, PPARgamma, SREBP and CREB associated programs play a role in regulating genes involved in fatty acid uptake, storage and oxidation [48, 49]. These regulatory processes enable tumour cells to adapt their lipid metabolism in response to changes in extracellular fatty acid concentrations. Our data are consistent with such metabolic regulatory changes, although the specific upstream signalling pathways responsible for the observed changes need to be investigated further.

The expression of FASN, the key enzyme of de novo lipogenesis, was downregulated in CRC cells upon uptake of exogenous fatty acids. Fitting to elevated circulating fatty acid levels in obese patients, a previous study found CRCs with no or only weak FASN expression were more frequently found in patients with high BMI compared with patients with normal BMI [50]. Correlating to this, the expression of ACLY, which is upregulated in tumour cells compared to non‐tumorous cells to fuel lipogenesis, was lower in the presence of elevated fatty acid levels in the medium of CRC cells.

Together, these findings suggest that elevated extracellular fatty acid concentrations may alter the balance between endogenous lipid synthesis and exogenous lipid utilization. This metabolic flexibility may be relevant under conditions in which extracellular fatty acids availability is increased.

In addition to in vitro analysis, we studied PLIN2 expression as a surrogate marker for lipid droplets in tumorous tissues of CRC patients with normal BMI and elevated BMI indicating obesity. PLIN2 is a key component for lipid droplet biogenesis [51]. PLIN2 overexpression has been shown to be associated with increased lipid droplet biogenesis and triglyceride content, although its expression alone should not be interpreted as a direct quantitative measure of lipid droplet numbers. Nevertheless, it is described as a biomarker for lipid droplet formation in several tissues [52]. Fitting to the in vitro data and our hypothesis that hyperlipidemia favours the uptake of exogenous lipids from the circulation and the formation of lipid droplets, we observed higher PLIN2 expression in obese CRC patients compared to CRC patients with normal weight, although this difference did not reach significance. Nevertheless, we found a significant correlation between PLIN2 expression and CPT1A expression in human CRC tissues. Certainly, these findings need to be validated in larger and independent cohorts of CRC patients. Furthermore, the BMI can only serve as an indirect marker since it describes the metabolic status and does not directly reflect circulating free fatty acid concentrations or systemic hyperlipidemia. Thus, functional studies would be required to prove the elevated uptake of serum lipids into the tumorous tissue of CRC patients. Still, our data give a further indication that obesity‐associated hyperlipidemia can promote CRC progression and indicate that PLIN2 might have potential as a prognostic marker in CRC patients, although our in vivo results lacked significance and require further evaluation but provide a basis for future mechanistic studies and in vivo studies. Future studies focusing on detailed metabolic phenotyping and circulating lipid measurements are required to determine whether tumour lipid metabolism differs according to metabolic status.

Besides uptake of elevated FAs from the circulation by tumour cells, there are further mechanisms by which dietary uptake of fatty acids and hyperlipidemia can affect cancer progression. High‐fat diets induce a gut microbial dysbiosis, metabolomic dysregulation and gut barrier dysfunction [53]. Consecutively, elevated endotoxin levels in the circulation promote cancer development and progression [54]. Furthermore, fat‐mediated alterations of the gut microbiota lead to an altered bile acid metabolism and activation of the bile acid receptor farnesoid X receptor (FXR), which promotes CRC development and progression [55]. Moreover, it has been described that hyperlipidemia can lead to a metabolic reprogramming of natural killer cells and herewith limited antitumor responses [56]. Furthermore, hyperlipidemia may have an impact on obesity‐associated therapy resistance as described in different cancer entities including CRC [57]. Thus, antiangiogenetic drugs have been shown to induce the expression of CD36, a critical transporter for the uptake of fatty acids into different tumour cells including CRC [58, 59]. Furthermore, antiangiogenetic therapies induce hypoxia and beta‐oxidation as well as desaturation of fatty acids by SCD1 require oxygen. Thus, hypoxic cancer cells rely on a higher uptake of exogenous (unsaturated) fatty acids [59, 60, 61]. Therefore, it might be speculated whether obesity or hyperlipidemia, respectively, have an impact on the efficacy of antiangiogenic therapy in CRC.

### 4.1 Conclusion

Besides de novo lipogenesis and lipolysis, uptake of exogenous fatty acids is the third route by which cancer cells can fuel their high demand for fatty acids. Our in vitro data indicate that the route of the exogenous uptake could be particularly important in obesity associated hyperlipidemia and herewith, may also be a further critical factor by which obesity promotes CRC development and progression. Accordingly, our findings suggest a potential association between PLIN2 and metabolic alterations in CRC. Larger and independently validated cohorts incorporating more comprehensive metabolic parameters beyond BMI, such as circulating free fatty acids concentrations, are required to determine its clinical relevance. Furthermore, the here provided model mimicking elevated extracellular fatty acids for CRC cells in vitro could help to unravel the molecular mechanism of fatty acid uptake, lipid droplet formation and the involvement of lipid droplets in promoting the tumorigenicity of CRC cells. A better understanding of these processes might contribute to the development of new strategies for cancer prevention and therapy.

## Author Contributions

**Tatjana Itzenhäuser:** investigation, writing – original draft, methodology, data curation, formal analysis, visualization, writing – review and editing. **Fulya Suzan Schildt:** investigation, methodology, visualization, writing – review and editing, formal analysis, data curation. **Seraphine Jochem:** investigation, writing – review and editing, methodology, visualization, formal analysis. **Judith Sommer:** investigation, methodology, validation, writing – review and editing. **Michael Stürzl:** writing – review and editing, methodology, data curation, supervision, investigation. **Elisabeth Naschberger:** investigation, methodology, writing – review and editing, formal analysis, data curation. **Anja Katrin Boßerhoff:** conceptualization, writing – original draft, writing – review and editing, resources, supervision, validation, investigation. **Claus Hellerbrand:** writing – original draft, conceptualization, investigation, funding acquisition, methodology, validation, visualization, writing – review and editing, project administration, supervision, data curation, resources.

## Funding

This work was supported by Deutsche Forschungsgemeinschaft (HE2458) and IZKF Erlangen (D42).

## Conflicts of Interest

The authors declare no conflicts of interest.

## Supporting information


**Supplementary Table 1.** Molecular background of colorectal cancer cell lines [27].

**Supplementary Figure 1.** Effect of oleate treatment on perilipin expression. HT29, HCT116, LoVo, SW480 and SW48 were incubated with 0.4 mM oleate for 24 h. (A) PLIN2 mRNA and protein expression analysed by quantitative RT‐PCR and western blot. (B) PLIN1, PLIN2, PLIN4 and PLIN5 mRNA expression of HT29 cells analysed by quantitative RT‐PCR (C) ACLY protein expression of HT‐29 and Caco‐2 (*p < 0.05 compared to control (Ctr)).
**Supplementary Figure 2**. Effect of oleate treatment on key enzyme of lipid metabolism in HCT116, LoVo, SW480 and SW48. (A) ACLY mRNA expression. (B) FASN mRNA expression. (C) SCD1 mRNA expression. (D) DGAT2 mRNA expression analysed by quantitative RT‐PCR. (*p < 0.05 compared to control (Ctr)).
**Supplementary Figure 3**. Effect of oleate treatment on beta oxidation and lipolytic enzymes.
Colon cancer cells were incubated with indicated oleate (OL) concentrations for 24 h. (A) CPT1A mRNA and protein expression was analysed. (B) Mito Stress test with prior etomoxir injection. Basal and maximal respiration of HT29 and Caco‐2 cells. (C) ATGL protein expression of indicated CRC cells analysed by western blot. (D) HSL and MAGL mRNA expression levels of HT29 cells (*p < 0.05 compared to control (Ctr)).
**Supplementary Figure 4**. Effect of cellular lipid accumulation on tumorigenicity of cancer cells. HCT116, LoVo, SW480 and SW48 cells were treated with 0.4 mM OL for 24 h. (A) Proliferation and (B) migratory activity of CRC cells (*p < 0.05 compared to control (Ctr)).
**Supplementary Figure 5**. Effect of cellular lipid accumulation on expression of EMT markers. HCT116, LoVo, SW480 and SW48 were treated with indicated OL concentrations for 24 h. mRNA expression of EMT‐markers (A) E‐CAD and (B) N‐CAD and (C) TWIST and (D) SNAIL (*p < 0.05 compared to control (Ctr)).
**Supplementary Figure 6**. Effect of oleate uptake on IL8 expression and PLIN2 expression in tumorous tissue of patients with colorectal carcinoma. (A) IL8 mRNA expression of HCT116, LoVo, SW480 and SW48 analysed by RT‐qPCR. (B) Correlation between high/low PLIN2 expression and disease free survival of CRC patients applying a Sveen‐Skotheim‐Colon‐GSE24549‐GPL11028 dataset of 83 patients and SurvExpress database (*p < 0.05 compared to Ctr). (C) Correlation between high/low CPT1 expression and disease free survival of CRC patients applying a Smith Beauchamp Colon GSE17536 dataset of 145 patients and SurvExpress database (*p < 0.05).

## Data Availability

All data generated in this study are included in the article and the Supporting Information files S1 and S2 and will be made available upon reasonable request.
