# Supplementary material for: Lipid Accumulation Promotes Tumorigenic Behaviour of Colorectal Cancer Cells
Source: J Cell Mol Med. 2026 Oct 3;30(19):e71393. doi: 10.1111/jcmm.71393 (PMC13634124; doi:10.1111/jcmm.71393)
Supplement: Supplementary file 1 [file JCMM-30-e71393-s001.pdf]

Supplementary Figure 1

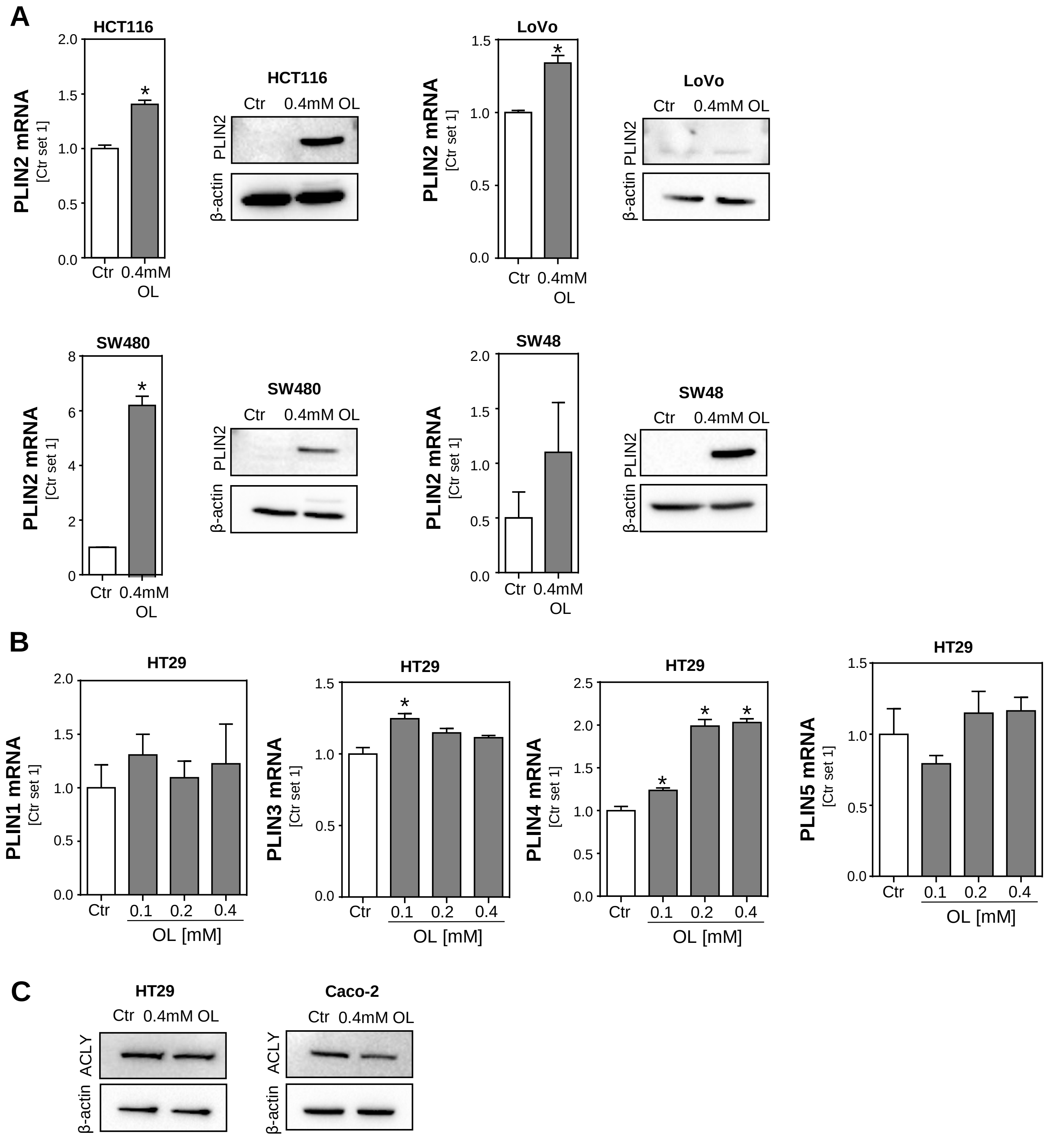

Supplementary Figure 1: Effect of oleate treatment on perilipin expression.
HT29, HCT116, LoVo, SW480 and SW48 were incubated with 0.4 mM oleate for 24h. **(A)** PLIN2 mRNA and protein expression analyzed by quantitative RT-PCR and western blot. **(B)** PLIN1, PLIN2, PLIN4 and PLIN5 mRNA expression of HT29 cells analyzed by quantitative RT-PCR **(C)** ACLY protein expression of HT-29 and Caco-2. (*: $p < 0.05$ compared to control (Ctr)).

Supplementary Figure 2

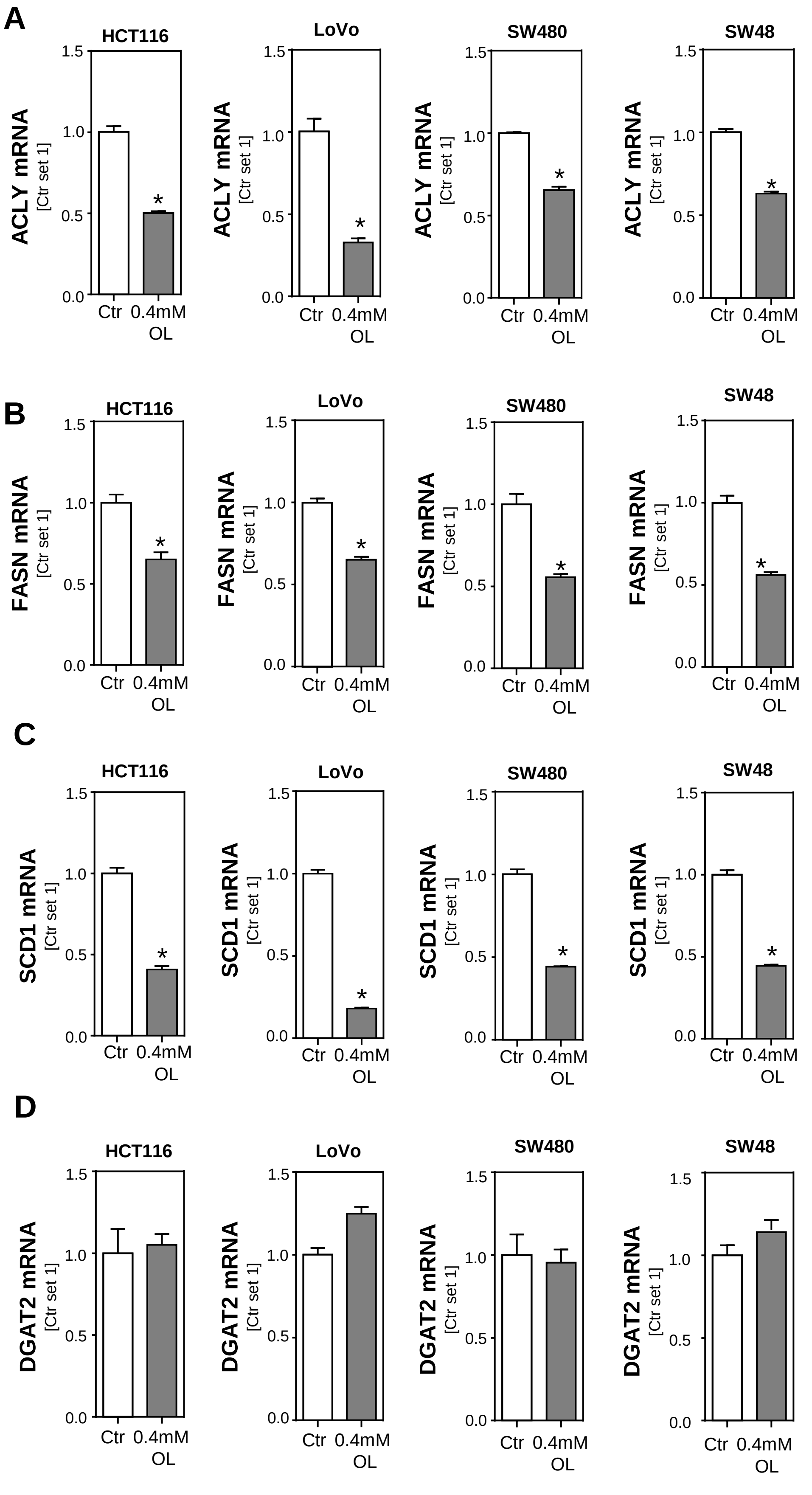

Supplementary Figure 2: Effect of oleate treatment on key enzymes of lipid metabolism in HCT116, LoVo, SW480, SW48.
Colon cancer cells were incubated with indicated oleate (OL) concentrations for 24h. **(A)** ACLY mRNA expression. **(B)** FASN mRNA expression. **(C)** SCD1 mRNA expression. **(D)** DGAT2 mRNA expression analyzed by quantitative RT-PCR. (*: $p < 0.05$ compared to control (Ctr))

Supplementary Figure 3

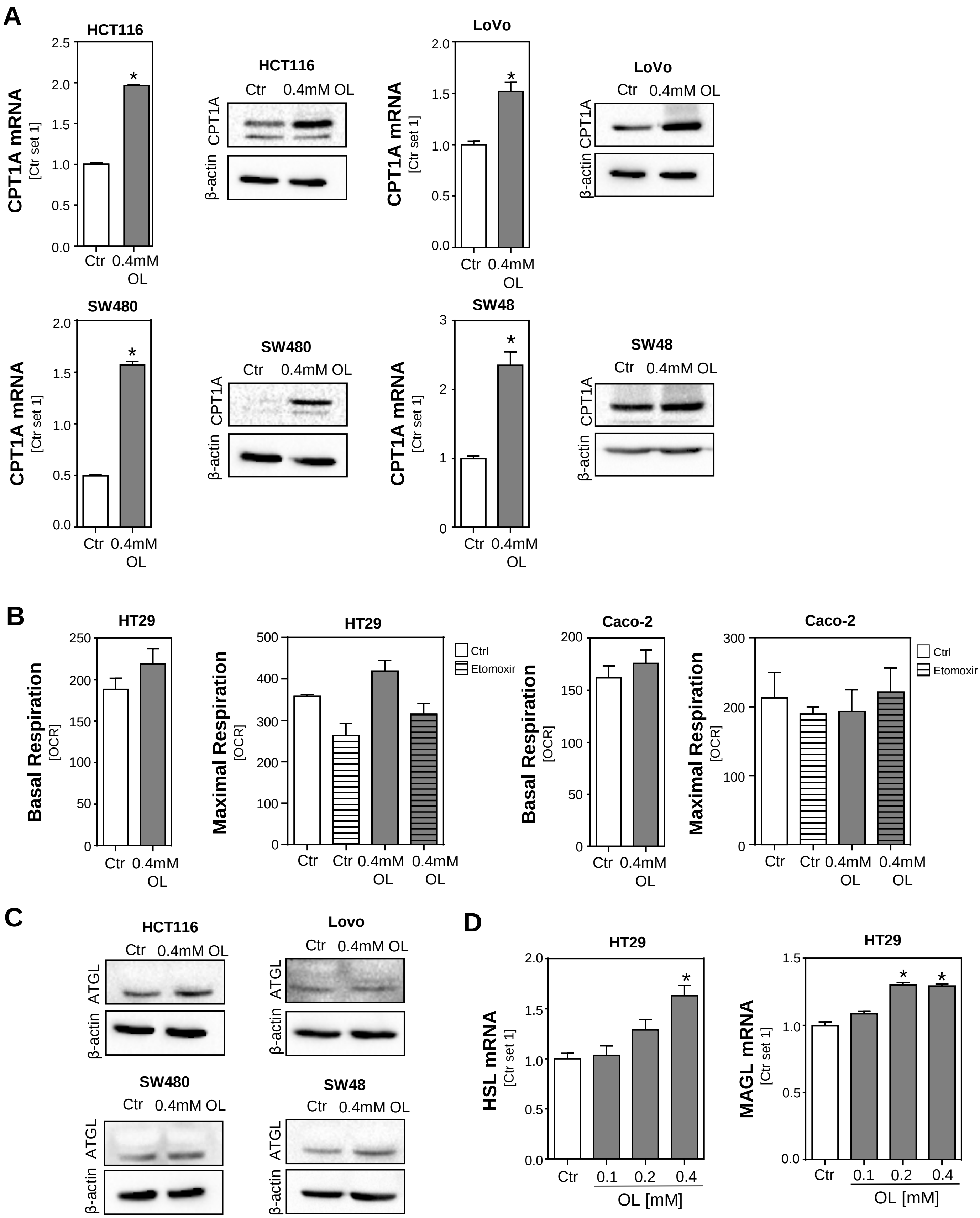

Supplementary Figure 3: Effect of oleate treatment on beta oxidation and lipolytic enzymes. Colon cancer cells were incubated with indicated oleate (OL) concentrations for 24h. **(A)** CPT1A mRNA and protein expression was analyzed. **(B)** Mito Stress test with prior etomoxir injection. Basal and maximal respiration of HT29 and Caco-2 cells. **(C)** ATGL protein expression of indicated CRC cells analyzed by western blot. **(D)** HSL and MAGL mRNA expression levels of HT29 cells. . (*: $p < 0.05$ compared to control (Ctr))

Supplementary Figure 4

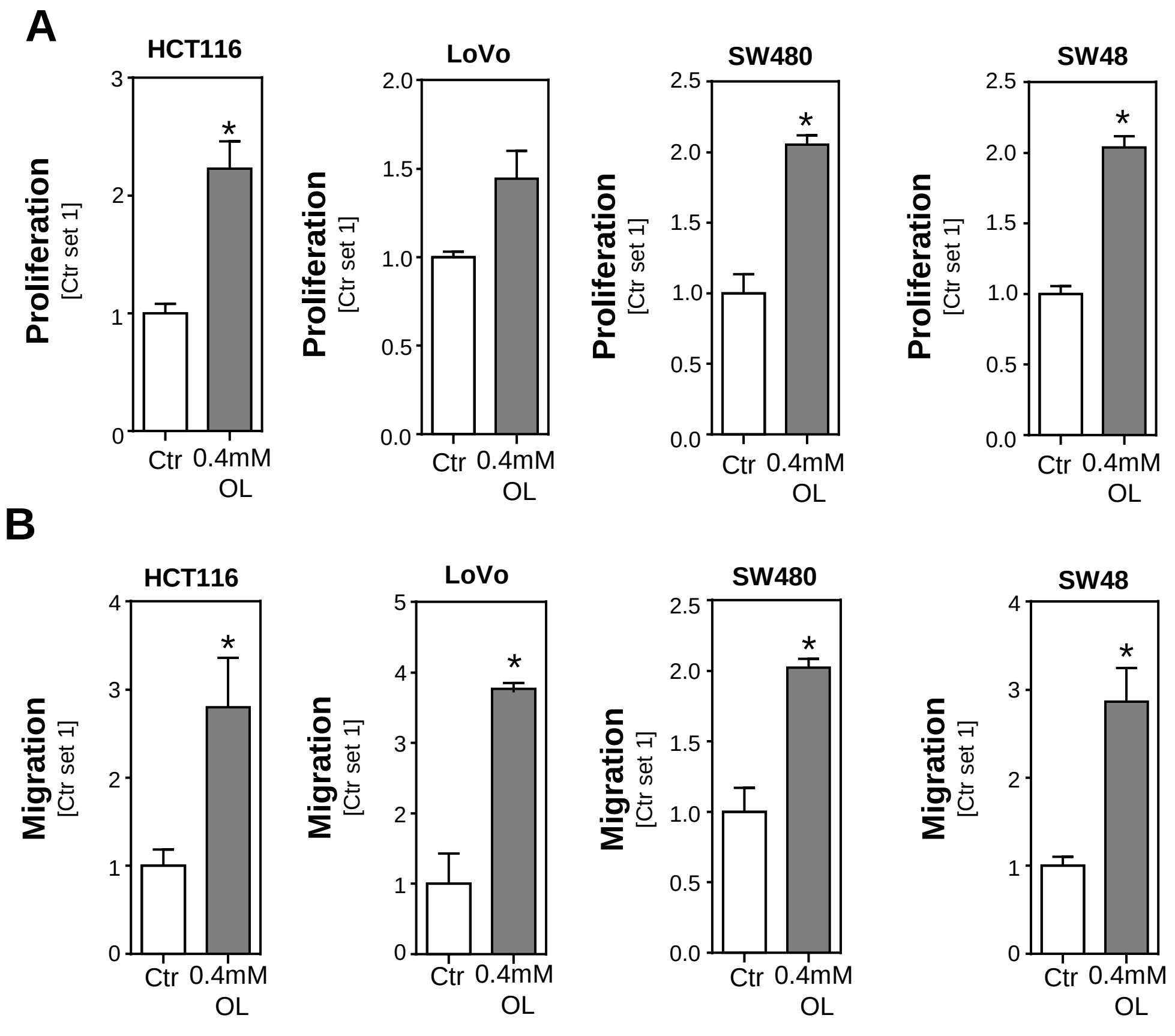

Supplementary Figure 4: Effect of cellular lipid accumulation on tumorigenicity of cancer cells.
HCT116, LoVo, SW480 and SW48 cells were treated with 0.4 mM OL for 24h. **(A)** Proliferation and **(B)** migratory activity of CRC cells (*: $p < 0.05$ compared to control (Ctr))

Supplementary Figure 5

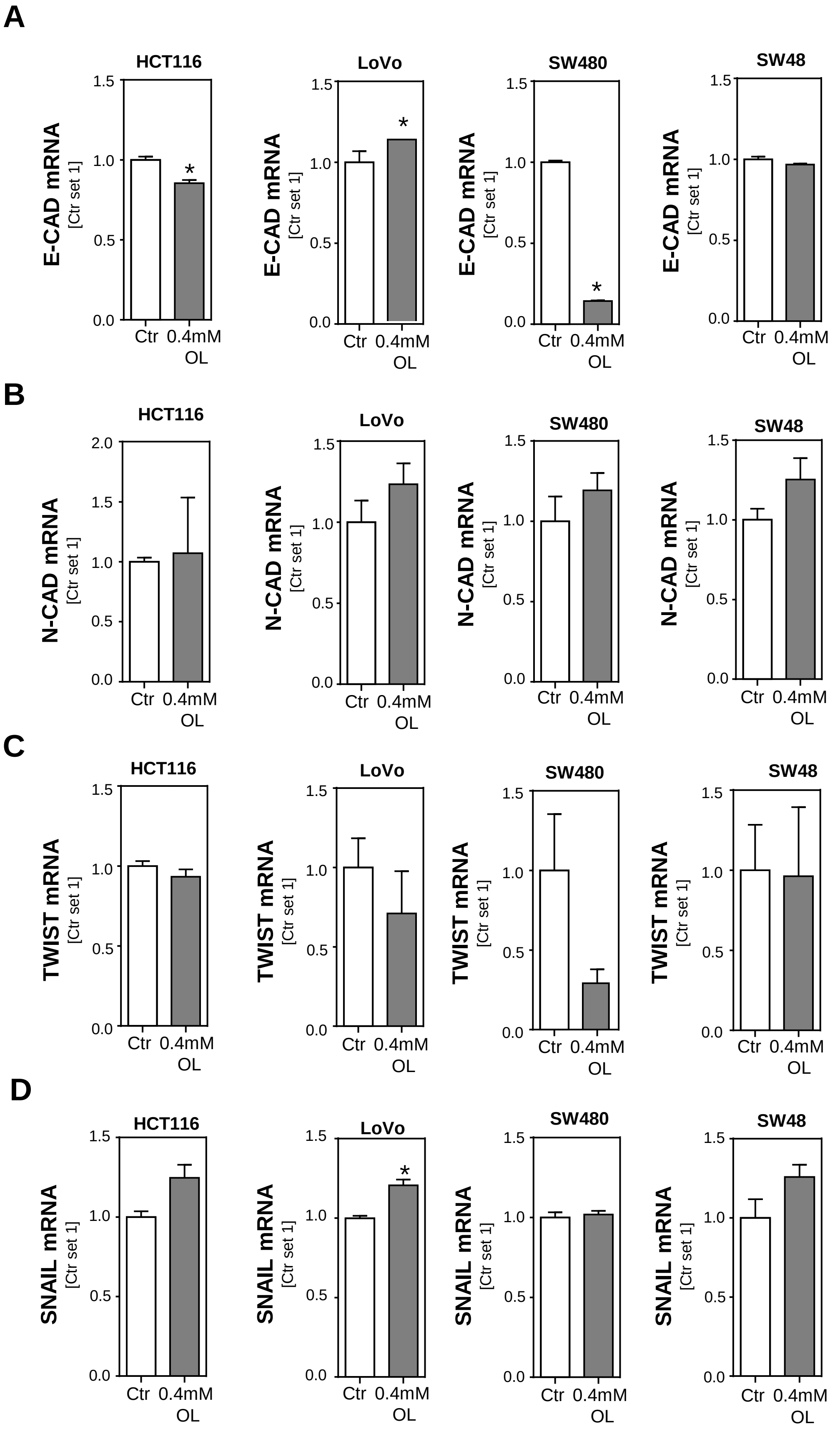

Supplementary Figure 5: Effect of cellular lipid accumulation on expression of EMT markers HCT116, LoVo, SW480 and SW48 were treated with indicated OL concentrations for 24h. mRNA expression of EMT-markers **(A)** E-CAD and **(B)** N-CAD and **(C)** TWIST and **(D)** SNAIL. (*: $p < 0.05$ compared to control (Ctr)

Supplementary Figure 6

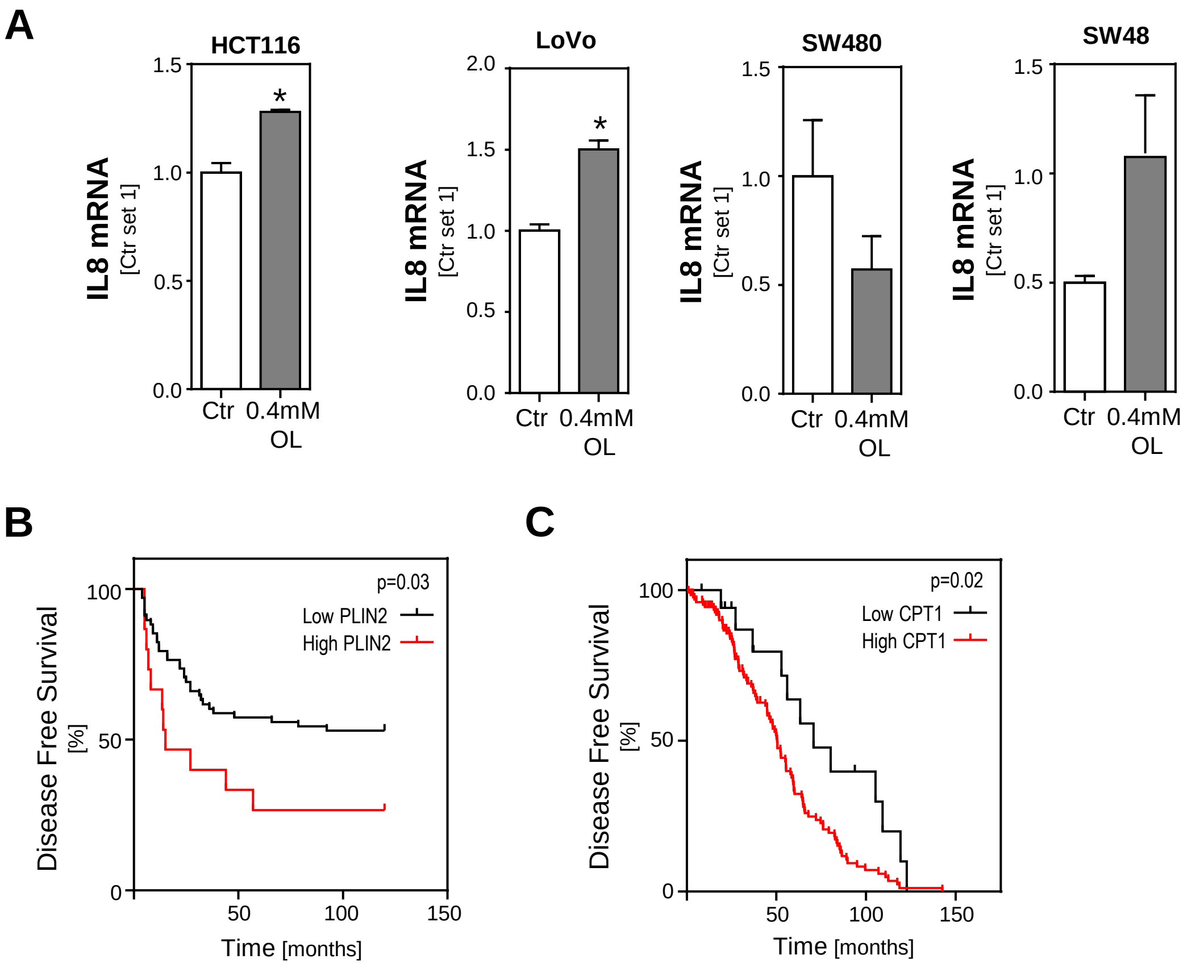

Supplementary Figure 6: Effect of oleate uptake on IL8 expression and PLIN2 expression in tumorous tissue of patients with colorectal carcinoma
(A) IL8 mRNA expression of HCT116, LoVo, SW480 and SW48 analyzed by RT-qPCR. **(B)** Correlation between high/low PLIN2 expression and disease free survival of CRC patients applying a Sveen-Skotheim-Colon-GSE24549-GPL11028 dataset of 83 patients and SurvExpress database. (*: $p < 0.05$ compared to Ctr). **(C)** Correlation between high/low CPT1 expression and disease free survival of CRC patients applying a Smith Beauchamp Colon GSE17536 dataset of 145 patients and SurvExpress database. (*: $p < 0.05$).
